# Clinical Ultrasound Applications in Obstetrics and Gynecology in the Year 2024

**DOI:** 10.3390/jcm13051244

**Published:** 2024-02-22

**Authors:** Florian Recker, Ulrich Gembruch, Brigitte Strizek

**Affiliations:** Department of Obstetrics and Prenatal Medicine, University Hospital Bonn, Venusberg Campus 1, 53127 Bonn, Germany; ulrich.gembruch@ukbonn.de (U.G.); brigitte.strizek@ukbonn.de (B.S.)

Ultrasound imaging stands as a fundamental technology in the realms of obstetrics and gynecology, utilizing high-frequency sound waves to create detailed images of the internal structures of the body [1]. This non-invasive, safe, and cost-effective imaging modality has revolutionized the fields of prenatal and gynecological care [2]. Its ability to provide real-time visualization has transformed diagnostic processes, allowing for immediate assessment and monitoring. The evolution of ultrasound technology, particularly with the advent of Doppler, three-dimensional (3D), and four-dimensional (4D) imaging, has expanded its scope of applications, thereby enhancing both diagnostic accuracy and the quality of patient care [3].

Ultrasound imaging operates on the principle of echolocation. High-frequency sound waves, when transmitted into the body by a transducer, are reflected back by internal tissues and organs. These reflected sound waves, or echoes, are then captured by the transducer and translated into visual images by a computer. The varying densities and compositions of bodily tissues result in different echo patterns, enabling the visualization of structures within the body, such as organs, blood vessels, and, in the case of obstetrics, the developing fetus [4].

In obstetrics, ultrasound is a vital tool throughout all stages of pregnancy. During the first trimester, it is indispensable for confirming intrauterine pregnancy, assessing the viability of the fetus, estimating gestational age, and performing nuchal translucency screening to evaluate the risk of chromosomal abnormalities such as Down syndrome. The second trimester brings the critical anatomy scan, where a detailed examination of the fetal anatomy is conducted to identify any structural anomalies. This scan also assesses the placental location and the volume of amniotic fluid, both of which are crucial for fetal well-being [5].

The third trimester focuses on fetal growth and well-being. Ultrasound during this phase is used to monitor the growth of the fetus, particularly in pregnancies deemed high-risk, such as those with gestational diabetes or hypertension. A biophysical profile, combining ultrasound imaging with fetal heart rate monitoring, is often performed to assess the health of the fetus.

Doppler ultrasound, a specialized form, is employed to evaluate the blood flow in fetal and placental vessels. This is particularly important in cases where there is a concern for conditions such as fetal growth restriction or placental insufficiency.

The introduction of 3D and 4D ultrasound technologies has further revolutionized obstetric imaging. These advanced forms of ultrasound provide detailed, three-dimensional images of the fetus, offering an unprecedented view of fetal anatomy that aids in the diagnosis of certain abnormalities. Additionally, 4D ultrasound, which shows real-time fetal movements, has added a new dimension to prenatal care, enhancing the bonding experience for expectant parents and providing a unique opportunity for early interaction with the fetus [6].

In the field of gynecology, ultrasound serves as a key diagnostic tool for a variety of conditions. It is routinely used to evaluate the uterus, ovaries, and fallopian tubes, helping in the diagnosis and management of conditions such as uterine fibroids, ovarian cysts, and endometriosis. Ultrasound is pivotal in fertility assessments, where it aids in monitoring follicular development and guiding procedures like egg retrieval in assisted reproductive technologies.

This modality is also fundamental in the evaluation of pelvic pain, a common symptom in gynecological practice. It helps in the diagnosis of conditions such as ectopic pregnancy, pelvic inflammatory disease, and ovarian torsion. In the realm of gynecologic oncology, ultrasound serves as an initial assessment tool for cancers of the reproductive organs, although its findings are often supplemented with more comprehensive imaging techniques for accurate staging and management.

While ultrasound is a versatile and invaluable tool in obstetrics and gynecology, it is not without its limitations. The quality and accuracy of ultrasound imaging can be affected by factors such as operator skill, patient body habitus, and the presence of intervening structures like gas or bone. The technology also has limitations in terms of deep tissue penetration and may not always provide comprehensive information on certain pathologies, necessitating the use of complementary imaging modalities for a thorough evaluation [7].

The future of ultrasound imaging in obstetrics and gynecology is marked by continuous innovation and advancement. Emerging technologies and techniques, such as elastography and the integration of artificial intelligence (AI) for enhanced image analysis, are expected to overcome some of the current limitations and expand the capabilities of ultrasound imaging [8]. These advancements promise to further refine diagnostic accuracy and improve patient outcomes in these vital fields of medicine.

In gynecology, adnexal masses are frequently cited as a primary reason for undergoing ultrasonography (US) in the field of gynecology, primarily driven by the critical need for the early detection of ovarian cancer. Distinguishing between benign and malignant tumors is not only crucial for accurate diagnosis but also significantly impacts the direction of subsequent diagnostic procedures and therapeutic planning. In clinical practice, the examination of adnexal masses typically involves transvaginal US, which combines grayscale 2D images with color Doppler imaging to evaluate vascularization. However, the accurate identification and classification of adnexal masses presents substantial challenges, even for seasoned examiners. In response to these challenges, the International Ovarian Tumor Analysis (IOTA) group has devised US-based guidelines to aid in the classification of adnexal tumors.

In recent years, the field has seen a notable shift towards the automated analysis of US images of adnexal masses. This approach has been recognized for its potential to support diagnostic decision making by assisting both inexperienced and experienced examiners. From the 11 studies examined, research conducted between 2009 and 2023 highlighted the initial use of AI in sonographic assessment by the research group led by Amor et al., employing pattern recognition analysis for classifying adnexal masses within a new reporting system [9]. The majority of these studies analyzed 2D images, with a subset also incorporating color Doppler imaging to enhance diagnostic accuracy.

A significant focus of current research is the automated differentiation between benign and malignant tumors, with six studies dedicated to this endeavor. The application of AI in this context aims to improve diagnostic accuracy, reduce examination times, and facilitate early detection, which is particularly crucial in managing breast cancer. Breast US has been emphasized for its advantages, especially in women with dense breast tissue and in underserved areas, where it serves as a critical diagnostic and screening tool.

Despite the promising results, including AI’s ability to outperform average trained radiologists in some instances, challenges remain. These include the limitations imposed by small sample sizes for algorithm training, data homogeneity, absence of external validation, and the necessity for algorithms to consider clinical contexts. Moreover, studies have indicated potential issues with AI interpretation, such as the misinterpretation of metastases or secondary ovarian cancer due to their rare representation in datasets and differences in clinical presentation.

Beyond adnexal masses, AI’s role in evaluating the endometrium, pelvic floor, and other gynecological conditions like endometriosis, premature ovarian failure, uterine fibroids, follicle tracking, and ectopic pregnancies has been explored. Each area presents unique challenges but also demonstrates the potential for AI to enhance diagnostic accuracy, efficiency, and patient care. For instance, the application of AI in assessing endometrial thickness and texture, and in the management of pelvic floor dysfunction, shows promise in improving patient outcomes through more precise and quicker diagnostics.

Ultrasound imaging remains an indispensable tool in the field of obstetrics and gynecology, continually evolving to offer safe, cost-effective, and detailed visualization of the female reproductive system and developing fetus. Its extensive applications, ranging from routine prenatal screenings to complex gynecological evaluations, underscore its integral role in enhancing patient care and clinical outcomes [10]. As technology advances, ultrasound imaging is poised to overcome current challenges and further extend its capabilities, solidifying its position as a cornerstone in the realm of women’s health care.

In this Special Issue, selected subjects on new imaging applications and insights are presented.

In this context, Borboa-Olivares et al. present a study where they evaluated subcutaneous fat tissue in the fetuses of women with well-controlled diabetes using 3D ultrasound, and AI classifiers were investigated, finding larger fat areas in fetuses from diabetic mothers compared to a healthy control group. The study’s comprehensive classifier model, including variables like subcutaneous fat measure and maternal BMI, effectively predicted the impact of maternal diabetes on fetal subcutaneous fat, independent of fetal growth.

Danathan-Stumpf et al. show in a prospective study the effectiveness of a novel three-dimensional (3D) body scanner with MR pelvimetry for assessing pelvic dimensions in planning vaginal breech deliveries. Among 73 singleton pregnancies intended for vaginal breech birth, the study found that the 3D body scanner, particularly the ratio of waist girth to maternal height, was at least as effective as MRI in predicting successful vaginal delivery. However, further large-scale studies are needed to confirm these findings.

Walter et al. conducted a retrospective study over 20 years at a tertiary center, examining the course and outcome of 21 cases of fetuses diagnosed with primary cardiomyopathy (CM). They found a 40% overall survival rate, with genetic etiology confirmed in 50% of cases and prenatal isolated right ventricular involvement as a significant parameter for survival. The study suggests that while the prenatal detection of CM is feasible, it requires a classification method for improved consultation and management, noting a poor outcome in many cases but emphasizing that increased examiner awareness could influence optimal multispecialized care.

In this context, Pontones et al. conducted a study to assess the feasibility and acceptance of self-guided mobile ultrasound by pregnant women in routine prenatal care. The study included 46 women who used mobile ultrasound systems to examine fetal heartbeat, profile, and amniotic fluid, finding that while two-thirds could imagine performing the examination at home, most preferred professional support. The success rates for locating target structures varied, and the study concludes that while there is acceptance for self-examination, further research is needed to determine its role in prenatal care and its impact on fetomaternal outcomes.

Manzo and colleagues established two-dimensional ultrasonographic (2D-US) reference ranges for the normal fetal cerebellar area from 13 to 39 weeks of gestation. Analyzing 252 normal singleton pregnancies, they found a significant positive correlation between cerebellar area and gestational age, providing several 2D-US nomograms for the cerebellar area. The study suggests that understanding the typical dimensions of the fetal cerebellar area could aid in identifying cerebellar abnormalities and enhance the detection of posterior fossa anomalies in future prenatal assessments.

Luca et al. evaluate in a prospective study the effectiveness of strain ratio (SR) analysis at the internal cervical US in predicting spontaneous preterm birth (PTB). Involving 114 high-risk pregnant patients, the study found that SR, particularly when combined with other parameters, showed high accuracy in predicting PTB before 37 weeks of gestation, with even higher predictive accuracy for extremely preterm births before 28 weeks. The study concludes that SR is a promising tool for predicting PTB and warrants further evaluation in diverse patient cohorts.

Likewise, Moreno-Espinosa et al. evaluate the effectiveness of quantitative ultrasound lung texture analysis using quantusFLM^®^ version 3.0 (quantusFLM, Barcelona, Spain) for predicting neonatal respiratory morbidity (NRM) in twin pregnancies. Involving 166 cases between 27.0 and 38.6 weeks of gestation, the study found that quantusFLM^®^ predicted NRM with a sensitivity of 42.9%, specificity of 95.9%, and an accuracy of 89.2%. The study demonstrates the potential of this non-invasive method for predicting NRM in twin pregnancies, highlighting its high specificity, negative predictive value, and overall good performance.

Orlandi and colleagues report on a case of prenatal-diagnosed intrathoracic left kidney (ITK) associated with congenital diaphragmatic hernia (CDH), complemented by a systematic review of similar cases. Their findings, based on eleven documented cases, indicate that CDH is a rare cause of ITK, typically diagnosed around 29 weeks of gestation. The study highlights that prenatal diagnosis and counseling are crucial for planning effective prenatal and postnatal management, leading to favorable outcomes after surgical repair of the herniated kidney and associated CDH.

In comparison, Barbieri et al. conducted a systematic review of umbilical vein blood flow volume (UV-Q) reference ranges in uncomplicated pregnancies and compared these findings with data from a local cohort. Their research, which followed the PRISMA guidelines and included 15 datasets, revealed substantial heterogeneity among the reported UV-Q central values. However, they found that when using consistent sampling methodologies and formulae, UV-Q assessment is accurate and reproducible. This suggests potential clinical applications for UV-Q measurement in obstetric practice.

In this context, Pappalardo et al. describe their experience in the prenatal diagnosis of body stalk anomaly complicated by ectopia cordis, a severe defect where the heart is located outside the thorax, as part of first-trimester sonographic screening. They report two cases, diagnosed at 9 and 13 weeks of gestation, respectively, using high-quality two- and three-dimensional ultrasonographic images with the Realistic Vue and Crystal Vue techniques. Despite normal fetal karyotype and CGH-array results, both pregnancies were terminated due to the poor prognosis of this anomaly. This study highlights the importance of early diagnosis, achievable between 10 and 14 weeks of gestation, using advanced sonographic techniques.

Matlac et al. show a prospective, monocentric, single-arm study to investigate the safety and efficacy of using US-guided High-Intensity Focused Ultrasound (HIFU) for treating symptomatic uterine fibroids. As the only center in German-speaking countries utilizing this technology, their study aims to evaluate not only the clinical outcomes of HIFU, such as symptom relief and fibroid size reduction, but also its effects on laboratory parameters and the structural integrity of uterine tissue. This research addresses the gap in data regarding the impact of HIFU on these specific aspects.

Plöger et al. report on the successful intraoperative use of indocyanine green (ICG) dye for assessing ovarian perfusion in a 17-year-old patient with ovarian torsion who underwent ovary-preserving surgery. Their case, supported by a systematic literature review, indicates that ICG angiography is a feasible and safe technique in the surgical treatment of ovarian torsion, with potential implications for reducing the need for oophorectomy, although further research is needed to confirm these findings.

The overview by Jost et al. provides a systematic literature review on the applications of artificial intelligence (AI) in obstetrics and gynecology (OB/GYN) ultrasound imaging. AI-assisted ultrasound applications in OB/GYN encompass fetal biometry, echocardiography, neurosonography, as well as the identification of adnexal and breast masses, and the assessment of the endometrium and pelvic floor. While AI shows promise in automating plane acquisition, measurements, and pathology detection, this review emphasizes the need for further research in emerging and experimental fields within OB/GYN ultrasound imaging to harness the full potential of AI technology.

In this Special Issue, various aspects of prenatal and obstetric care have been explored, leveraging advanced technologies and innovative approaches. This comprehensive examination has been made possible through the judicious utilization of cutting-edge technologies and the application of pioneering methodologies. The collective body of work presented in these studies exemplifies the relentless pursuit of enhanced knowledge and improved practices in the field of maternal–fetal healthcare. In summary, this compilation of studies represents a testament to the relentless pursuit of excellence in maternal–fetal healthcare. Through the judicious amalgamation of cutting-edge technologies and innovative methodologies, these endeavors collectively contribute to the continuous enhancement of prenatal and obstetric care, paving the way for improved maternal and fetal outcomes.
